# Pain thresholds in elderly individuals: a cross-sectional observational study of the influence of gender and chronic non-cancer pain^☆^

**DOI:** 10.1016/j.bjane.2025.844665

**Published:** 2025-07-29

**Authors:** Áquila Lopes Gouvêa, Pedro Adde Anuardo, João Paulo Consentino Solano, Ângela Maria Sousa, Hazem Adel Ashmawi

**Affiliations:** a Divisão de Enfermagem do Instituto Central do Hospital das Clínicas da Faculdade de Medicina da Universidade de São Paulo, São Paulo, SP, Brazil; b AMA/UBS Integrada Jardim São Jorge, São Paulo, SP, Brazil; c Centro Universitário São Camilo, São Paulo, SP, Brazil; d Departamento de Cirurgia da Faculdade de Medicina da Universidade de São Paulo, São Paulo, SP, Brazil; e Divisão de Anestesia do Hospital das Clínicas da Faculdade de Medicina da Universidade de São Paulo, São Paulo, SP, Brazil; f Departamento de Anestesiologia da Faculdade de Ciências Médicas da Universidade Estadual de Campinas, Campinas, São Paulo, SP, Brazil

**Keywords:** Aging, Chronic pain, Elderly, Gender, Pain measurement, Pain threshold

## Abstract

**Background:**

The older population is growing, and it is estimated that, by 2050, people aged 60-years or more will have reached two billion. The increased life expectancy has led to a higher incidence of chronic degenerative diseases, contributing to increased pain complaints. This study aims to compare the pain threshold after mechanical stimulation in older adults according to gender and presence or absence of chronic pain and find the prevalence and intensity of chronic pain in this population.

**Methods:**

This was a cross-sectional observational study with a convenience sample in the outpatient clinic at two research centers. All participants answered sociodemographic and clinical questionnaires, and the Pressure Pain Threshold (PPT) was assessed with an algometer. Patients reporting chronic pain answered the Geriatric Pain Measure (GPM) questionnaire.

**Results:**

The sample consisted of 230 individuals, aged 60 to 96 years, 67.8% women and 32.2% men. Chronic pain prevalence was 47.8%, 29.7% in men and 56.4% in women. PPT was significantly lower in women (4.49 ± 1.78 kg) than in men (6.41 ± 1.92 kg). PPT in older individuals presenting chronic pain (4.58 ± 1.93 kg) was lower than in older individuals without chronic pain (5.58 ± 2.01 kg). There was no significant difference between genders in pain assessment by GPM.

**Conclusions:**

Pressure pain threshold was lower in older women and in patients with chronic pain, the association between gender and lower pain threshold was stronger than observed with chronic pain.

## Introduction

Aging of the world population is an evident and fast-paced phenomenon, and it is expected that, by 2050, the number of people aged 60 years or more reaches two billion.^1^ Changes resulting from aging lead to increased incidence of chronic degenerative diseases, contributing to pain complaints.^2^

Pain exhibits variability according to gender and sex and, usually, women report more intense, frequent and persistent pain symptoms than men,^3^ which has been confirmed in epidemiological studies of prevalence and characteristics of chronic pain.^4^^,^^5^ These differences between women and men are influenced by genetic, social, psychological and hormonal factors.^5^

Changes in gonadal hormone levels as a result of aging play an important role in pain sensitivity and tolerance. Reduced levels of estrogen in women (postmenopausal) and testosterone in older men may influence pain perception.^6^ Gonadal hormones are related to increased pain perception in animals.^7^ In older women, in menopause, when female gonadal hormones have decreased secretion, there are questions about how women behave in relation to pain.^8^, ^9^, ^10^

Studies show that aging appears to alter the pain threshold.^11^^,^^12^ With aging, sensitivity to pain is reduced, confirming that pain thresholds increase with age,^13^ with men having a higher pain threshold for mechanical stimuli. However, there are no studies addressing the influence of chronic pain in conjunction with gender in older people.

We hypothesized that pain threshold after mechanical stimulation in older women would be similar to that of older men due to decreased gonadal hormones in menopausal women. This study aimed to compare pain threshold after mechanical stimulation in older individuals according to gender and presence or absence of chronic pain and establish the prevalence and intensity of chronic pain in the population.

## Methods

This was a cross-sectional observational study with convenience sample. The study was approved by the Ethics Committee for Analysis of Research Projects of Hospital das Clínicas da Faculdade de Medicina da Universidade de São Paulo (#1.751.968) and registered at clinicaltrials.gov (NCT06855797).

The study was carried out in two geriatric outpatient centers: Geriatric Outpatient Clinic of the Central Institute of the Hospital das Clínicas da Faculdade de Medicina da Universidade de São Paulo, and Geraldo de Paula Souza School Health Center of the Faculdade de Saúde Pública da Universidade de São Paulo, centers serving geriatric outpatients.

People aged 60 years or more were considered older adults, according to the definition used in Brazil and recommended by the World Health Organization for developing countries.^14^ Although chronic pain is defined as pain that persists or recurs for more than 3 months,^15^ this study included patients presenting pain for at least 6 months, and the data collection instrument adopted was the measurement of pain in geriatric patients (Geriatric Pain Measure-GPM).^16^

### Inclusion criteria

Individuals aged 60 years or more without cognitive impairment assessed using the Mini Mental State Examination (MMSE) were included. Cutoff values varied according to education, as follows: 20 for illiterate individuals, 25 for 1- to 4 years of schooling, 26 for 5 to 8 years of schooling, 28 for 9 to 11 years of schooling, and 29 for more than 11 years of schooling.^17^

### Exclusion criteria

Participants presenting cognitive difficulty understanding and answering the questionnaires or with cancer pain were excluded.

### Data collection

Data collection lasted for the period necessary to reach the number of subjects calculated for the study (07/2017 to 12/2019).

After signing the informed consent form, participants were interviewed. All participants answered the Questionnaire for Assessment of Sociodemographic and Clinical Characteristics. Patients reporting chronic pain for six months or more answered the Geriatric Pain Measure (GPM) questionnaire, and pain threshold was assessed by mechanical stimulus.

Pressure pain thresholds were assessed using a pressure algometer (Push Pull Force Gauge – Model 12-0304), which measures force exerted between 1 and 10 kg.cm^-2^. The device was applied on the right or left Trapezius muscle, on the suprascapular portion of the muscle, then pressure was applied, and participants reported the moment they felt pain or discomfort. Each subject was tested three times with 5 min intervals and the mean of the three measurements was recorded as the pressure pain threshold.

### Sample size

Sample size was calculated as 230 patients, assuming a standard deviation of 2.0 points on the pain threshold scale,^18^ with an expected allocation of one man for every two women, with 90% power to detect an average difference of 1 point on the pain threshold scale at a significance level of 5%.

### Statistical analysis

Quantifiable variable values were described by mean and standard deviation, minimum and maximum values. Variables were summarized and segmented by gender and presence of chronic pain. Categorical variables were compared by Fisher's exact test and, for continuous variables, by Student's *t*-test, when normal distribution was present. Otherwise, variables were compared using the non-parametric Mann-Whitney test.

The assessment of the primary outcome, pressure pain threshold, was performed using the linear regression model considering the interaction between gender and chronic pain. Sensitivity analyses adjusted for age and interactions with gender and pain were also performed. Analysis was performed using the R4.0.2 program (R Core Team, 2020). The significance level in the statistical tests was 5%.

## Results

Two hundred and eighty-three individuals were eligible for the study, forty-seven declined to participate in the study, two were excluded for not meeting minimum MMSE scores, and four opted out of the study. Two hundred and thirty subjects were assessed, 67.8% (n = 156) women and 32.2% (n = 74) men. The participants’ average age was 75.8 years, 76.2 years in women and 74.9 years in men. Participants aged 60 – 74 years were the most frequent, 45.7% (n = 105), and older individuals aged 75 – 84 years, 37.8% (n = 87). The youngest participant was 60 years old and the oldest participant was 96 years-old (Table 1).

**Table 1:** Sample distribution according to gender and age.

Parameter	Male (n = 74)	Female (n = 156)	Total (n = 230)	p-value
Age (mean ± SD)	74.9 ± 8.4	76.2 ± 8.0	75.8 ± 8.2	0.234^a^
Age group				
60 – 74 years	37 (50%)	68 (43.6%)	105 (45.7%)	0.665^b^
75 – 84 years	26 (35.1%)	61 (39.1%)	87 (37.8%)	
85 years or more	11 (14.9%)	27 (17.3%)	38 (16.5%)	

a Student’s *t*-test.

b Fisher's exact test. * Significance level: p < 0.05. Values are presented as mean ± standard deviation or number (%).

The most frequent morbidities in the sample were: systemic arterial hypertension, 49.1% (n = 113); diabetes mellitus, 22.6% (n = 52), and osteoarthritis, 13.5% (n = 31). Chronic pain prevalence in the sample was 47.8% (n = 110), 56.4% (n = 88) among women and 29.7% (n = 22) among men. Chronic pain was about twice more frequent in women than in men (56.4% vs. 29.7%, p < 0.001) (Table 2). Chronic pain intensity, using GPM score, was not different between men and women, 42.8 ± 24.4 in men and 51.0 ± 25.6 in women, meaning moderate pain (Table 2). Prevalent sites of pain were lower limbs, 58.2% (n = 64); lumbar region, 40.9% (n = 45), and shoulders 38.2% (n = 42).

**Table 2:** Sample distribution according to gender and chronic pain prevalence, and Geriatric Pain Measure according to gender.

Parameter	Gender			p-value
	Male (n = 74)	Female (n = 156)	Total (n = 230)	
Chronic pain				
Yes	22^c^ (29.7)	88^c^ (56.4%)	110 (47.8%)	< 0.001^a^
No	52^c^ (70.3%)	68^c^ (43.6%)	120 (52.2%)	
Pain assessment GPM	(n = 22)	(n = 88)	(n = 110)	
GPM Total (0‒100)	42.8 ± 24.4	51.0 ± 25.6	49.4 ± 25.5	0.18^b^

a Fisher's exact test.

b Student’s *t*-test.

c Significance level: p < 0.05. Values are presented as mean ± standard deviation or number (%).

**Figure 1:**
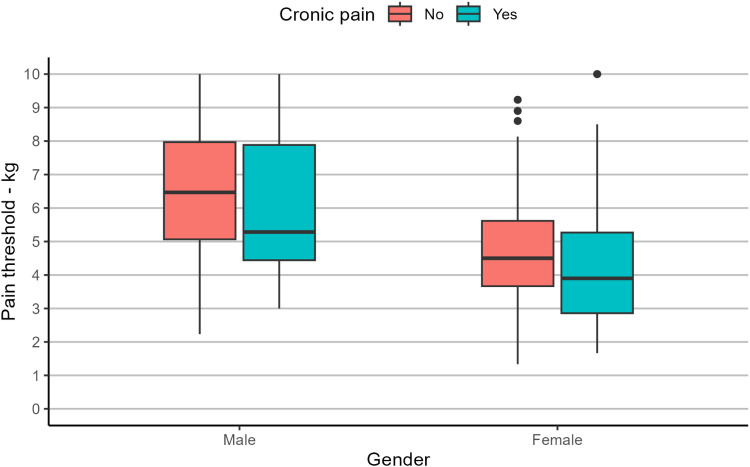
Mean pressure pain threshold between genders and chronic pain.

PPTs found in women (4.49 ± 1.78 kg.cm^-2^) were lower than in men (6.41 ± 1.92 kg.cm^-2^) (p < 0.001) (Table 3). Pain thresholds in chronic pain participants (4.58 ± 1.93 kg.cm^-2^) were lower than in participants without chronic pain (5.58 ± 2.01 kg.cm^-2^) (p < 0.001), (Fig. 1, Table 3).

**Table 3:** Sample distribution according to mean pressure pain threshold, gender and presence or absece of chronic pain.

Variables	Number of participants (n = 230)	PPT (kg.cm^-2^)	p-value^a^
Gender			
Male	74	6.41 ± 1.92	< 0.001
Female	156	4.49 ± 1.78	
Chronic pain			
No	120	5.58 ± 2.01	< 0.001
Yes	110	4.58 ± 1.93	

a Student’s *t*-test. PPT, Pressure Pin Threshold (kg.cm^-2^). Values are presented as mean ± standard deviation.

The interaction between female gender and chronic pain was assessed, since pain was more prevalent in women, and a linear regression model for PPT according to gender and presence of chronic pain showed no interaction effect (p = 0.82) (Table 4). Another linear regression model verified the independent association of gender, presence of chronic pain with pain threshold. It was estimated that women had an average value of 1.76 kg.cm^-2^ lower pain threshold than men (95% CI 1.24–2.28, p < 0.01). Chronic pain also reduced pain threshold by an average of 0.59 kg.cm^-2^ (95% CI 0.11–1.07, p = 0.02) (Supplementary Material). Female gender was more associated to lower pressure pain threshold than chronic pain. There was no difference in pressure pain threshold and estimates of gender and chronic pain according to age stratification (Supplementary Material).

**Table 4:** Linear regression model for pain threshold according to gender and chronic pain interaction.

Factor	Coefficient	Standard error	*t*-value	p
Intercept	6.56	0.25	26.10	< 0.01
Female	−1.72	0.33	−5.14	< 0.01
Chronic pain	−0.50	0.46	−1.09	0.28
Interaction Gender (F): Pain (Yes)	−0.12	0.55	−0.23	0.82

² = 21.6%.

## Discussion

This cross-sectional study compared pressure pain threshold between older men and women with or without chronic pain and assessed the prevalence of chronic pain in older outpatients.

PPT in older adults was different between males and females, and in those with or without chronic pain. Our initial hypothesis was that older women in menopause, without the influence of female gonadal hormones, would have a pain threshold similar to men, due to the absence of the pronociceptive effects of gonadal hormones. However, the pain threshold was, on average, 1.76 kg.cm^-2^ lower in women than in men. Similarly, patients with chronic pain had decrease in pain threshold of 0.59 kg.cm^-2^ compared to those without chronic pain. Both female gender and chronic pain were associated to lower pain thresholds; however, interestingly, being female was more associated to lower pain threshold than presenting chronic pain.

Testosterone, present in males, can contribute with a protective or analgesic effect of the hormone,^9^^,^^10^ while social, psychological or cultural factors also influence pain perception in females, with women seemingly presenting more catastrophizing and less self-efficacy behaviors, leading to increased pain perception.^19^ Seemingly, decreased gonadal female hormones are less important than gender.

Although findings on this issue are controversial, our results were similar to previous studies on PPT between genders in healthy older individuals without chronic pain, with a lower pain threshold in women than in men.^11^^,^^13^ One small study (n = 40) showed no difference between older men and women; however, the study has no report of the number of older men and women in the study.^12^ Pain thresholds after heat are also lower in older women compared to older men,^20^ and pain thresholds for different pain stimuli may also be lower in women.

As in most studies involving older adults, most subjects were female (67.8%) and the prevalence of chronic pain in this population was 47.8%, similarly to other studies on community-dwelling older individuals, which range from 28.7% to 60.4%.^21^, ^22^, ^23^

Although chronic pain prevalence was higher in women than in men, the characterization of chronic pain according to GPM was not different between the genders, with pain intensity showing moderate intensity in both genders, similarly to a previous study.^16^ Prior to this study, GPM had not been used for comparisons between genders, and there are still no elements to state that the instrument is adequate to assess the role of gender on intensity of pain in older adults.

This study has some limitations due to its adoption of a convenience sample of participants, which may have introduced selection bias, sometimes overrepresenting certain patient profiles, creating difficulty in applying the results to the general older population. Due to this being a cross-sectional study, we can only show the association but not causality between gender, chronic pain and lower PPT.

We found no previous study of older individuals presenting chronic pain. An interesting and intriguing result was the lower association of previous chronic pain compared to gender on pain threshold. Older women showed higher decrease in pain threshold than older individuals presenting chronic pain. It has been well established that individuals with chronic pain, for example, chronic low back pain, have lower PPT than healthy individuals.^24^ Chronic pain would be expected to impact pain threshold more than gender. It is difficult to consider this result without further studies to confirm and explain this finding and, in our opinion, it is still early to apply these findings to clinical practice, in treatments of acute and chronic pain in the older population; however, gender and chronic pain will probably be addressed in the future for customized pain treatments.

## Conclusion

Gender was the main factor associated with decreased pressure pain threshold. PPT was lower in older women than in older men. Chronic pain was also associated with lower pain threshold, which was lower in people presenting chronic pain, and PPT was lower in women than in individuals with chronic pain. Chronic pain was more prevalent in older women.

## Funding

The authors have no funding sources to declare for this manuscript.

## Conflicts of interest

The authors declare no conflicts of interest.

## Appendix Supplementary materials

Image, application 1

## Footnotes

Supplementary material associated with this article can be found in the online version at doi:10.1016/j.bjane.2025.844665.
