# Supplementary material for: Pain thresholds in elderly individuals: a cross-sectional observational study of the influence of gender and chronic non-cancer pain
Source: Braz J Anesthesiol. 2025 Jul 29;75(5):844665. doi: 10.1016/j.bjane.2025.844665 (PMC12446678; doi:10.1016/j.bjane.2025.844665)
Supplement: Supplementary file 1 [file mmc1.docx]

**BJAN-D-24-00567**

**Supplementary Material**

Sample distribution according to gender, race, marital status.

| **Variables** | **Male (n = 74)** | **Female (n = 156)** | **Total (n = 230)** | **p^a^** |
| --- | --- | --- | --- | --- |
| Race |  |  |  |  |
| White | 61^b^ (82.4%) | 105^b^ (67.3%) | 166 (72.2%) | 0.017 |
| Black | 5^b^ (6.8%) | 18^b^ (11.5%) | 23 (10%) |  |
| Brown | 1^b^ (1.4%) | 19^b^ (12.2%) | 20 (8.7%) |  |
| Yellow | 7 (9.5%) | 12 (7.7%) | 19 (8.3%) |  |
| Indigenous | 0 (0%) | 2 (1.3%) | 2 (0.9%) |  |
| Marital status |  |  |  |  |
| Married | 43^b^ (58.1%) | 50^b^ (32.1%) | 93 (40.4%) | 0.001 |
| Widowed | 14^b^ (18.9%) | 59^b^ (37.8%) | 73 (31.7%) |  |
| Single | 10 (13.5%) | 28 (17.9%) | 38 (16.5%) |  |
| Divorced | 7 (9.5%) | 10 (6.4%) | 17 (7.4%) |  |
| Separated | 0^b^ (0%) | 8^b^ (5.1%) | 8 (3.5%) |  |
| Consensual union | 0 (0%) | 1 (0.6%) | 1 (0.4%) |  |

^a^ Fisher's exact test.

^b^ Significance level: p < 0.05.

Values are presented or number (%).

Sample distribution according to gender, education, professional status and economic classification.

| **Variables** | **Male (n = 74)** | **Female (n = 156)** | **Total (n = 230)** | **p^a^** |
| --- | --- | --- | --- | --- |
| Education (years) |  |  |  |  |
| Over 11 years | 39^b^ (52.7%) | 40^b^ (25.6%) | 79 (34.3%) | < 0.001 |
| 9 ‒ 11 years | 18 (24.3%) | 39 (25%) | 57(24.8%) |  |
| 1 ‒ 4 years | 12^b^ (16,2%) | 40^b^ (25.6%) | 52 (22.6%) |  |
| 5 ‒ 8 years | 5^b^ (6.8%) | 31^b^ (19.9%) | 36 (15.7%) |  |
| Illiterate | 0 (0%) | 6 (3.8%) | 6 (2.6%) |  |
| Current professional status |  |  |  |  |
| Retired | 62 (83.8%) | 123 (78.8%) | 185 (80.4%) | < 0.001 |
| Housewife | 0^b^ (0%) | 19^b^ (12.2%) | 19 (8.3%) |  |
| Employee | 11^b^ (14.9%) | 6^b^ (3.8%) | 17 (7.4%) |  |
| Self-employed | 0 (0%) | 6 (3.8%) | 6 (2.6%) |  |
| Unemployed | 1 (1.4%) | 1 (0.6%) | 2 (0.9%) |  |
| Sick leave | 0 (0%) | 1 (0.6%) | 1 (0.4%) |  |
| Economic classification |  |  |  |  |
| B2 | 26 (35.1%) | 45 (28.8%) | 71 (30.9%) | 0.304 |
| C1 | 18 (24.3%) | 38 (24.4%) | 56 (24.3%) |  |
| C2 | 9 (12.2%) | 38 (24.4%) | 47 (20.4%) |  |
| B1 | 9 (12.2%) | 16 (10.3%) | 25 (10.9%) |  |
| A | 8 (10.8%) | 10 (6.4%) | 18 (7.8%) |  |
| D‒E | 4 (5.4%) | 9 (5.8%) | 13 (5.7%) |  |

^a^ Fisher's exact test.

^b^ Significance level: p < 0.05.

Values are presented or number (%).

Locations of pain in elderly people with chronic pain.

| **Pain region** | **Total (n = 110)** |
| --- | --- |
| Lower limbs | 64 (58.2%) |
| Lumbar region | 45 (40.9%) |
| Shoulders | 42 (38.2%) |
| Hip | 32 (29.1%) |
| Upper limbs | 31 (28.2%) |
| Twin region | 27 (24.5%) |
| Feet | 23 (20.9%) |
| Cervical region | 20 (18.2%) |
| Face | 12 (10.9) |
| Abdominal | 8 (7.3%) |
| Thoracic | 7 (6.4%) |
| Cephalic | 5 (4.5%) |
| Generalized pain | 3 (2.7%) |

The total sum exceeds 100% because there are elderly people with pain in multiple locations.

Values are presented or number (%).

Sample distribution according to gender and associated diseases.

| **Variables** | **Male (n = 74)** | **Female (n = 156)** | **Total (n = 230)** | **p^a^** |
| --- | --- | --- | --- | --- |
| Associated Diseases |  |  |  |  |
| High blood pressure | 35 (47.3%) | 78 (50%) | 113 (49.1%) | 0.778 |
| Diabetes mellitus | 17 (23%) | 35 (22.4%) | 52 (22.6%) | 1 |
| Arthrosis | 5 (6.8%) | 26 (16.7%) | 31 (13.5%) | 0.041 |
| Depression | 4 (5.4%) | 8 (5.1%) | 12 (5.2%) | 1 |
| Vascular diseases | 3 (4.1%) | 8 (5.1%) | 11 (4.8%) | 1 |
| Peripheral arterial disease | 7 (9.5%) | 2 (1.3%) | 9 (3.9%) | 0.006 |
| Stroke | 2 (2.7%) | 4 (2.6%) | 6 (2.6%) | 1 |
| Asthma | 2 (2.7%) | 2 (1.3%) | 4 (1.7%) | 0.596 |
| Parkinson's disease | 0 (0%) | 4 (2.6%) | 4 (1.7%) | 0.308 |
| Obesity | 1 (1.4%) | 3 (1.9%) | 4 (1.7%) | 1 |
| Peptic ulcer | 1 (1.4%) | 2 (1.3%) | 3 (1.3%) | 1 |
| Chronic obstructive pulmonary disease | 1 (1.4%) | 1 (0.6%) | 2 (0.9%) | 0.541 |
| Anemia | 0 (0%) | 1 (0.6%) | 1 (0.4%) | 1 |
| Acquired Immunodeficiency Syndrome | 0 (0%) | 1 (0,6%) | 1 (0,4%) | 1 |
| Another disease | 1 (1,4%) | 8 (5,1%) | 9 (3,9%) | 0,278 |

^a^ Fisher's exact test.

The total sum exceeds 100% because there are elderly people within multiple diseases.

Values are presented or number (%).

Linear regression model for pain threshold according to gender and chronic pain.

| **Factor** | **Coefficient** | **standard error** | ***t*-value** | **p** |
| --- | --- | --- | --- | --- |
| Intercept | 6.58 | 0.22 | 29.58 | < 0.01 |
| (Feminine gender) | -1.76 | 0.26 | -6.69 | < 0.01 |
| (Chronic pain) | -0.59 | 0.25 | -2.40 | 0.02 |

R² = 21.6%.

**Sociodemographic Characteristics**

**1.Identification:**

Initials:______________ Date of birth: _____/____/_____

Gender: 1. Masculine ( ) 2. Feminine ( )

**2.Color or race -** White ( ) Black ( ) Yellow ( ) Mixed race ( ) Indigenous ( )

3. **Marital status** - A. Single ( ) B. Married ( ) C. Consensual union ( ) D. Divorced ( )

E. Separated ( ) F. Widowed ( )

4. **Do you live alone?** Yes ( ) 2. No ( )

5. **Religion -** A. Atheist ( ) B. Catholic ( ) C. Evangelical ( ) D. Spiritualist ( ) E. Buddhist ( )

F. No religion ( ) G. Others ( ) ________________

5.**1 Practitioner**:

A. Yes ( ) B. No ( )

6. **Domicile -** A. Owned ( ) B. Rented ( ) C. Other situation ( ) _______________________

7. **Employment Situation**

7.1. Profession _____________7.2. Main occupation___________7.3. Current occupation___________________ A. Employed ( ) B. Unemployed ( ) C. Retired ( ) D. On sick leave ( ) E. Housewife ( ) F. Self-employed ( ) G. Informal ( ) H. Other ( ) ___________

**Clinical Data**

1. Do you have any known diseases? 1. Yes ( ) 2. No ( ) If yes, which diseases?

A. Diabetes Mellitus ( ) B. High Blood Pressure ( ) C. Stroke ( ) I. COPD ( ) H. Asthma ( )

D. Coronary Artery Disease ( ) E. Vascular Diseases ( ) F. Pressure Ulcers ( )

G. Anemia ( ) J. Peptic Ulcer ( ) L. Osteoarthritis ( ) M. Obesity ( ) N. Epilepsy ( )

O. Depression ( ) P. Parkinson's Disease ( ) Q. Acquired Immunodeficiency Syndrome ( ) R. Limb Amputation ( )

Others: ________________________________

2. Are you taking hormone replacement therapy? ( ) Yes ( ) No If yes, which ones?_________________________________

**Pain threshold**

1st measurement_______________

2nd measure_______________

3rd measure_______________

**Chronic pain**

Have you felt any pain or aches that bothered you or bother you frequently in the last 6 months or more?

Yes ( ) No ( ) Pain duration ( ) months or ( ) years?___________Pain diagnosis?_________

Flow diagram I.

Flow diagram II.
